# sRNATargetDigger: A bioinformatics software for bidirectional identification of sRNA-target pairs with co-regulatory sRNAs information

**DOI:** 10.1371/journal.pone.0244480

**Published:** 2020-12-28

**Authors:** Xinghuo Ye, Zhihong Yang, Yeqin Jiang, Lan Yu, Rongkai Guo, Yijun Meng, Chaogang Shao

**Affiliations:** 1 College of Life Sciences, Huzhou University, Huzhou, Zhejiang, P.R. China; 2 Shanghai Institute of Plant Physiology and Ecology, Chinese Academy of Sciences, Shanghai, P.R. China; 3 College of Life and Environmental Sciences, Hangzhou Normal University, Hangzhou, Zhejiang, P.R. China; National Institute of Plant Genome Research (NIPGR), INDIA

## Abstract

Identification of the target genes of microRNAs (miRNAs), *trans*-acting small interfering RNAs (ta-siRNAs), and small interfering RNAs (siRNAs) is an important step for understanding their regulatory roles in plants. In recent years, many bioinformatics software packages based on small RNA (sRNA) high-throughput sequencing (HTS) and degradome sequencing data analysis have provided strong technical support for large-scale mining of sRNA-target pairs. However, sRNA-target regulation is achieved using a complex network of interactions since one transcript might be co-regulated by multiple sRNAs and one sRNA may also affect multiple targets. Currently used mining software can realize the mining of multiple unknown targets using known sRNA, but it cannot rule out the possibility of co-regulation of the same target by other unknown sRNAs. Hence, the obtained regulatory network may be incomplete. We have developed a new mining software, sRNATargetDigger, that includes two function modules, “Forward Digger” and “Reverse Digger”, which can identify regulatory sRNA-target pairs bidirectionally. Moreover, it has the ability to identify unknown sRNAs co-regulating the same target, in order to obtain a more authentic and reliable sRNA-target regulatory network. Upon re-examination of the published sRNA-target pairs in *Arabidopsis thaliana*, sRNATargetDigger found 170 novel co-regulatory sRNA-target pairs. This software can be downloaded from http://www.bioinfolab.cn/sRNATD.html.

## Introduction

Small RNAs (sRNAs) is a class of non-coding RNAs with lengths ranging from 18 to 30 nucleotides and different functions. In plants, some sRNAs are complementarily matched with specific bases of the target mRNAs, resulting in cleavage and degradation of the target transcripts. This kind of regulation can negatively modulate the expression of target genes, and it plays important roles in many biological processes, such as plant growth and development, disease resistance, and stress response [1, 2]. sRNAs can be classified according to their distinct characteristics into a variety of categories such as microRNAs (miRNAs) and *trans*-acting small interfering RNAs (ta-siRNAs).

Identifying and validating the target genes of these sRNAs are important steps for understanding their biological functions. sRNAs exert their effect by binding to the 3'-UTR of the target gene, and their function can be validated using reporter constructs, wherein genes such as luciferase are cloned upstream of the 3'-UTR of the target gene. The inhibitory effects of sRNAs on the target genes are quantified by comparing the changes in fluorescence values of in the presence or absence of sRNAs [3]. However, limited by the technical level, experimental cost, and experimental cycle, only a small number of sRNA-target pairs have been identified. Therefore, many computation-based sRNA-target prediction tools have been recently developed [4–6]. Making full use of the complementary property between sRNAs and their candidate targets, predictions can be achieved through large-scale sequence matching [7]. Although these results identify some genuine targets, false positive results may exist; thus, further experimental verification is required.

A large number of fragmented sequences captured by degradome sequencing can be mapped to the transcript sequences to find the signals for the enzymatic cleavages guided by specific sRNAs. Three types of high-throughput degradome sequencing platforms, *i*.*e*. parallel analysis of RNA ends (PARE) [8], genome-wide mapping of uncapped and cleaved transcripts (GMUCT) [9], and degradome sequencing [10] provide an efficient way to obtain degradation signals. Bioinformatics tools such as CleaveLand [11], SeqTar [12], PAREsnip2 [13], and sPARTA [14] make full use of the degradome data for sRNA-target prediction. This prediction is mainly achieved by performing the following steps: (1) Potential sRNA-target genes are screened based on sequence complementarity. (2) Specific cleavage sites on potential target genes are obtained based on the matching analysis of degradome signatures. The regulatory relationship is determined based on the correlation between the cleavage site and the binding site of sRNA to the target.

Currently, these tools are well used in different species and in different biological pathways [15–18]. It is well known that sRNA-target regulation is a complex biological process. The same mRNA may have multiple sRNA regulators, and the same sRNA may also have multiple downstream targets [19]. The software tools mentioned above can only predict the regulatory relationship between known sRNAs and mRNAs, which has the following problems: a) In addition to the known sRNAs, there may be other unknown sRNAs that co-regulate the target genes. If the regulatory role is attributed only to the known sRNA, it will lead to a big gap in the construction of the regulatory network. b) More importantly, if the expression levels of the unknown co-regulatory sRNAs are much higher than that of the annotated sRNA, the regulatory effect of this annotated sRNA on the predicted target might be replaced by the effects of the unknown sRNAs. Therefore, the key to highly reliable degradome-based identification of the sRNA-target relationship is to carefully consider the interference by unknown sRNAs.

We have previously proposed an approach for regulatory miRNA mining using a reverse mining method [20], which provides a feasible solution to the above problems. Once the target gene was predicted, all the potential sRNA regulators of this target could be extracted from sRNA high-throughput sequencing (HTS) data using the reverse mining method. The extent of the involvement of these sRNAs in target regulation can be determined according to their expression levels, so as to reliably identify most of the sRNAs that target a specific mRNA. Based on this idea, we improved the current degradome-based mining technology of sRNA-target genes, and developed a novel software named sRNATargetDigger for this purpose. sRNATargetDigger is easy to download and convenient to use, and it gives the user a good experience, as well.

## Materials and methods

### Data pre-processing

To reliably obtain more sRNA-target pairs, we need sRNA, mRNA, and degradome sequencing data. sRNA and degradome sequencing data were pre-processed. First, the adapter sequences were removed and the short sequence (including "N") whose bases cannot be determined was deleted. Next, the short sequence with a read count of 0 was also deleted, and reads per million (RPM) was used as the unit for normalization of the expression value of the sequence. The normalized count of an sRNA/degradome sequence = (the raw read count of the sequence/ the total read counts of the data set) × 10^6^.

### Candidate target gene prediction

In plants, sRNA and target genes show almost perfect base complementarity. A position-dependent scoring system based on the number of mismatches was used to judge the complementarity between an sRNA and its target gene(s). For example, the scoring rules proposed by Allen [21] and Fahlgren [22] have been used in many target gene prediction tools and models. According to the research results of Axtell *et al*. [23], the latest psRNATarget [7] tool included a new scoring rule to expand the seed sequence to 2–13 base pairs (bp), allowing only two mismatches (except G:U pairs), and a modified penalty score of the gap and final screening threshold. We screened potential candidate target genes of sRNAs from the cDNA database according to the psRNATarget scoring rules [7]:

- G:U pair,a penalty of 0.5 point will be imposed for each occurrence;
- There are no more than two mismatches (G: U matching is not included) from position 2–13 of 5'end of miRNA;
- For other nucleotide pairs that do not meet the Watson–Crick rule, a penalty of 1 point will be imposed for each occurrence. Except for a mismatch at positions 2–13, for each mismatch the score will be reduced by 0.5 points.
- In case of bulges, insertions, or deletions, a penalty of 2 points will be imposed for each occurrence; An additional 0.5 point penalty will be imposed for each nucleotide in the bulge consisting of more than one nucleotide; two bulges are allowed at most;
- Mismatches are not allowed in the tenth and eleventh positions;
- The total penalty points for a nucleotide of 19 bases in length were calculated, and those with no more than 5 penalty points were set as acceptable complementary sequences.

### Degradome-based identification of sRNA-target binding

The target gene cleavage signals caused by sRNA-guided cleavage could be detected using degradome sequencing. First, the degradome data were matched to the candidate target gene obtained in the previous step through a fast hash-table-based algorithm, and all of the 5' end site information that can be completely matched was recorded. Second, the signal intensity of the cleavage site and the signal intensity of the degraded background were calculated according to formula 1 and formula 2, respectively, and the sites with a ratio of both values 5 times or more were screened as specific cleavage sites.

Formula 1: The signal intensity of the cleavage site = the sum of counts of all sequences that are matched with degradomes at this site / the sum of the numbers of sequences that are matched with the degradomes at this site.

Formula 2: The signal intensity of the degraded background = the sum of counts of sequences that are matched with degradomes except at the cleavage site / the sum of the numbers of sequences that are matched with the degradomes.

**Fig 1:**
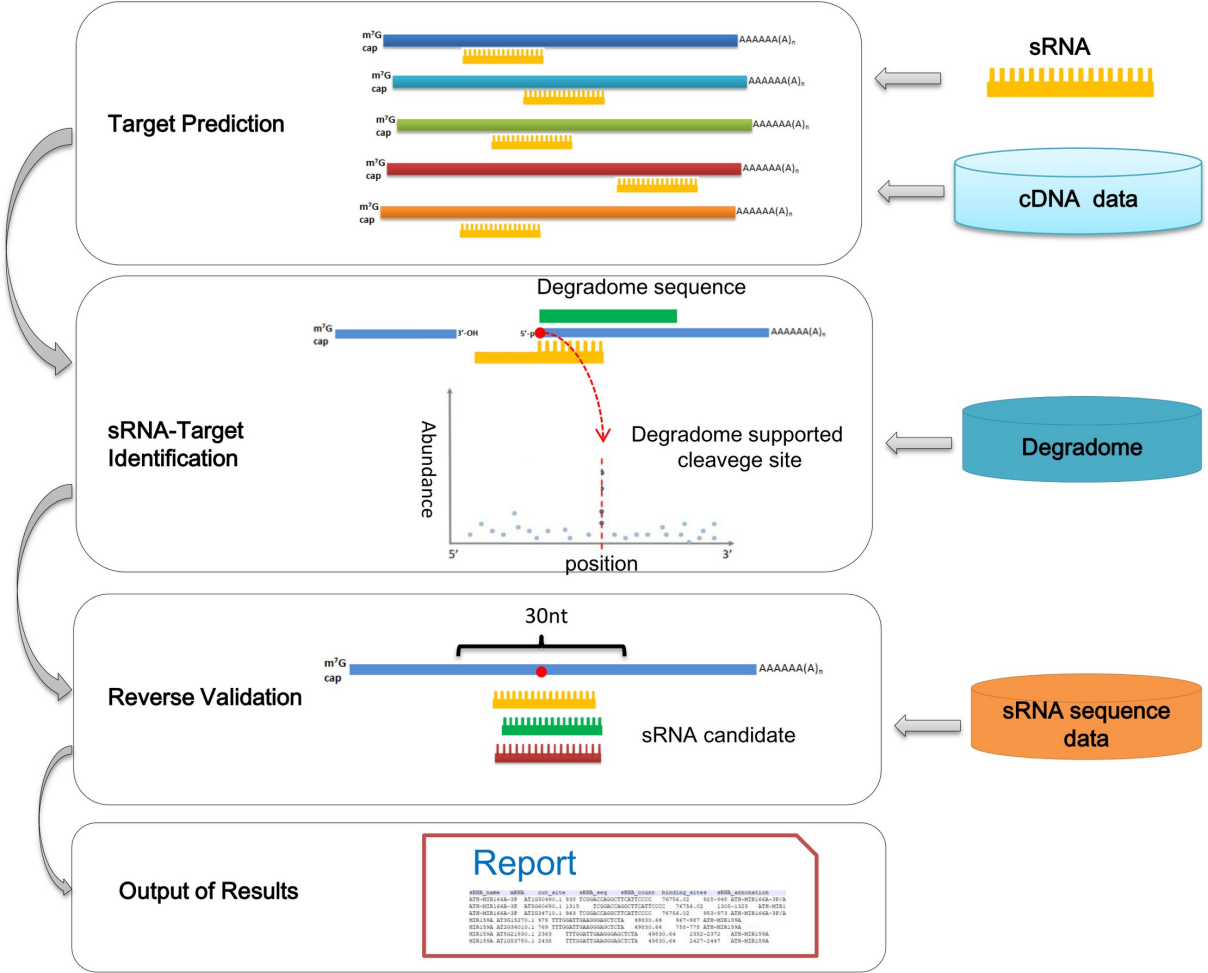
The workflow of the “Forward Digger” of sRNATargetDigger. The potential targets of the sRNA were screened out from cDNA data using sequence complementarity. Then, degradome sequences were mapped to these potential targets to identify the actual cleavage signals. Only when the cutting signal was within the middle of sRNA binding site, the sRNA-target pairs were retained. In the reverse validation step, 30-nt sRNA binding site extracted from the target was used as sequence complementary bait to search all possible regulatory sRNAs from the sRNA HTS data set. The sRNA-target regulatory relationship was confirmed if the known sRNA was identified again according to its expression levels.

According to the regulatory mechanisms of plant sRNAs and their specificities, when an mRNA sequence and the sRNA sequence satisfy the base complementarity characteristics, and the specific cleavage can be detected in the middle of sRNA binding site through the degradome, it is likely that there is a specific regulatory relationship between the mRNA and sRNA (Fig 1).

### Reverse verification of the sRNA-target regulatory relationship

Since one mRNA can be regulated by multiple sRNAs, it is necessary to perform further validation of the bound sRNA-target regulatory relationship to rule out the interference from other unknown sRNAs. According to the previous reports [6, 24–26], if the same site is regulated by multiple sRNAs, the cleavage effect at this site is largely accomplished by the qualified sRNA with the highest expression. Therefore, we took the cleavage site on the mRNA as the center and collected 30-nt sequences surrounding this site (15 nt upstream and downstream) as the candidate sRNA binding regions. Upon using the reverse mining technology, all the possible regulatory sRNAs were identified from the sRNA sequencing data set and sorted according to the expression levels of sRNAs. The sRNAs whose expression levels were less than 1/10 of the highest expression level were deleted. If a known sRNA was identified again, the sRNA-target regulatory relationship was considered true, and all co-regulatory sRNA information was outputted (Fig 1). If the known sRNAs were absent, this regulatory relationship was ignored.

### Software test

To test the reliability of the software tools, we used the degradome data, sRNA HTS data, and cDNA data stored in the public database to verify the sRNA-target regulatory relationship in *Arabidopsis*, which has been previously reported in the literature [7]. Since the expression patterns of many sRNAs and mRNAs were tissue-specific, we considered that this regulatory relationship was true as long as one tissue passed the “Forward Digger” or “Reverse Digger”function module. We downloaded sRNA and degradome HTS data from the GEO database (www.ncbi.nlm.nih.gov/geo/), comprising the *Arabidopsis* sRNA HTS data (GSM707682, GSM707683, GSM707684, GSM707685) and the degradome data (GSM278333, GSM278334, GSM278335 and GSM278370). The cDNA sequence information of *Arabidopsis* was downloaded from the TAIR10 (http://www.arabidopsis.org/).

The newly discovered co-regulatory sRNAs, which have not been reported in literature [7], were further tested using PAREsnip2 [13]. The parameters of PAREsnip2 were set as follows: " allow_mismatch_position_10 = true","allow_mismatch_position_11 = true"," core_region_start = 2", "core_region_end = 13", "max_mismatches_core_region = 2", "max_score = 5.5", and " max_gaps = 2".

## Results and discussion

### Architecture and features

**Fig 2:**
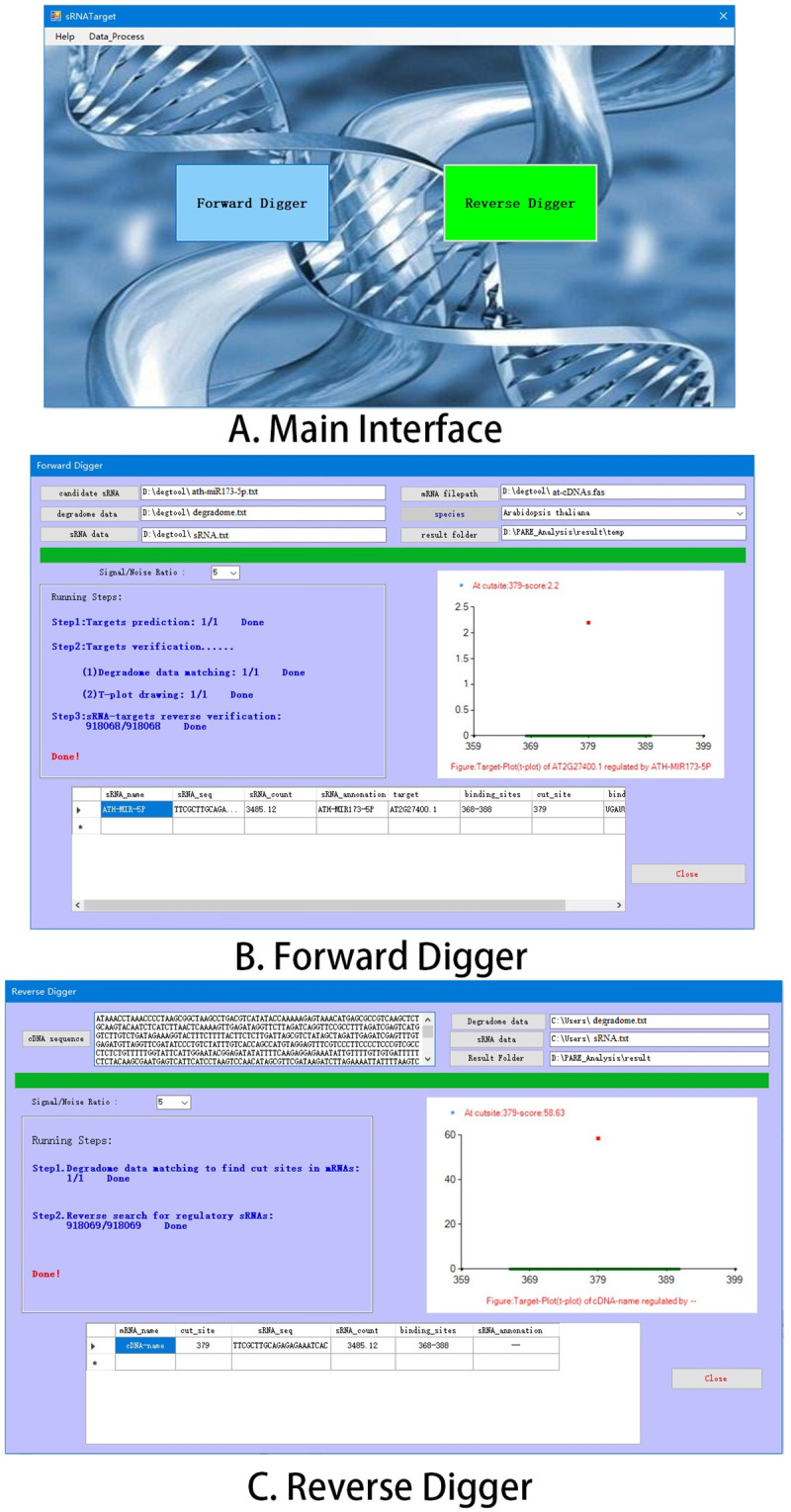
The software interface of sRNATargetDigger. A: Main interface. The regulatory relationship between the sRNA and the target mRNA can be identified bidirectionally by two modules, “Forward Digger” and “Reverse Digger”. B: “Forward Digger”. Candidate sRNA (regulatory sRNA) is used as query sequence to identify its regulatory target genes from cDNA data set and the co-regulatory sRNAs of the identified target genes will also be searched from sRNA HTS data set. C: “Reverse Digger”. The cDNA sequence (target gene) is used as query sequence to identify the regulatory sRNAs from the sRNA HTS data set according to the cleavage site on the target gene.

The sRNATargetDigger software used the degradome and sRNA HTS data to identify the potential sRNA-target regulatory relationship by including two function modules: “Forward Digger” and “Reverse Digger” (Fig 2A). Function of “Forward Digger”: sRNAs were known, and their regulatory target genes were identified from the cDNA data set. Function of “Reverse Digger”: *vice versa*, regulatory sRNAs were identified from the sRNA HTS data set according to the cleavage site on the target gene.

Users can click to enter the “Forward Digger” module (Fig 2B), which requires input of regulatory sRNA sequence, cDNA data set (for finding candidate target genes), degradome set (for finding specific cleavage sites on target genes), and sRNA HTS data set (used to verify the true expression of the regulatory sRNAs and find unknown co-regulatory sRNAs), and the users need to select the source species of the data from the species using the drop-down box. The correct selection of species can easily show in the results whether a certain sRNA has been annotated in the miRBase (release 22). In addition, users can modify result fold conveniently and save the results to corresponding folders on their computers. The above files that required to be inputted must be in FASTA format. After the input is completed, click the “Run” button, and the program will automatically run according to the analysis process shown in Fig 1. The user can track the running progress of the program in real time from the running prompt box on the left side of the interface. The “T-Plot” box on the right side of the interface displays the t-plot map matched with the degradome at the corresponding position of the corresponding gene, and then the final results are outputted in the text box below. The results are sorted by gene name and sRNA expression counts in descending order for easy reference. After the operation is completed, the users can find all the results in the result folder.

“Reverse Digger” is an improvement of our previous works [20]; it can reversely identify regulatory sRNAs based on the cleavage sites on the target genes (Fig 2C). The improvement was carried out mainly in the following two aspects: a) The original algorithm is limited to identifying miRNA. However, the other sRNAs (such as ta-siRNA) can also specifically cleave the target transcripts; “Reverse Digger” reversely identifies almost all these regulatory sRNAs. b) The original algorithm only provides an analysis process, and the users need to develop a program and use different software to complete the relevant analysis; “Reverse Digger” is an integrated pipeline that can automatically complete the analysis by inputting the formatted data, which is very convenient for most biological researchers.

### Software evaluation

We evaluated the sRNATargetDigger software on the sRNA-targets that have been reported in *Arabidopsis*. The corresponding benchmark data of *Arabidopsis* collected from the literature [7] included a total of 142 pairs of sRNA-target regulatory pairs. In plants, sRNAs are highly complementary to the target genes, and they often lead to the cleavage of the target gene mRNA by the Argonaute (AGO) protein; this specific cleavage can be detected using the degradome [27, 28]. We analyzed the regulatory relationship of 142 pairs of sRNA-targets. However, the results showed that no specific cleavage signal can be found using degradome in the middle of the sRNA binding site in 57 target genes (S1 Table). Therefore, the authenticity of the regulatory relationship in these 57 pairs of sRNA-targets needs to be further confirmed, and the possibility that their occurrence time was unmatched with the data set used for our verification could not be ruled out.

**Fig 3:**
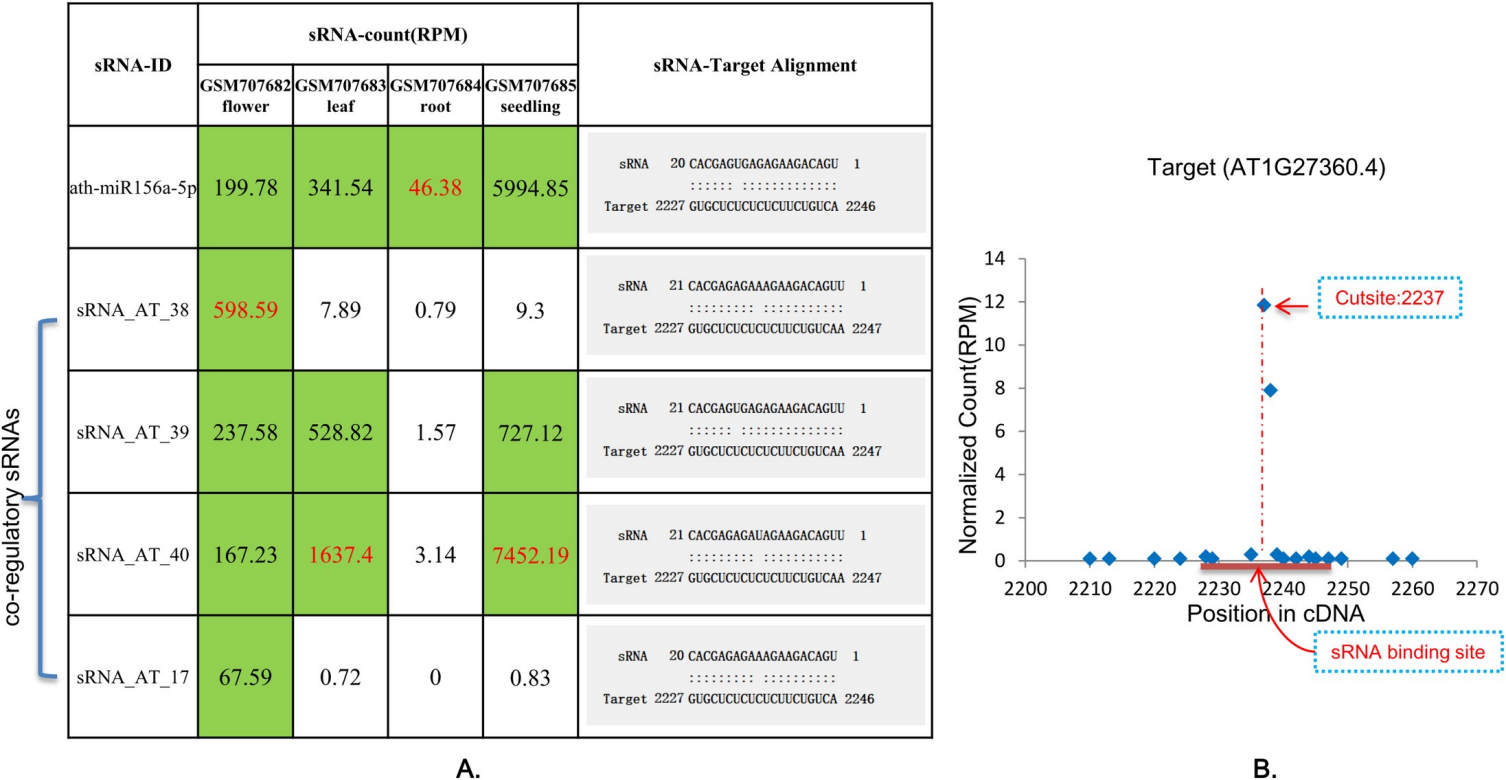
The miRNA ath-miR156a-5p and the co-regulating sRNAs with their target cDNA (AT1G27360.4) in *Arabidopsis*. A: Expression of ath-miR156a-5p and co-regulatory sRNAs in flowers, leaves, roots, and seedlings of *Arabidopsis*, and their alignments with target genes; green indicates that sRNAs played a regulatory role, white indicates that the regulatory role of the sRNA could be ignored; red number indicates that the sRNAs played the most important regulatory role. B: T-plot map obtained after matching of the target gene AT1G27360.4 and degradome. The abscissa indicates the position on the target gene, the ordinate indicates the intensity of the degradation signal, the red horizontal line indicates the sRNA binding site, and the red dotted line indicates the cleavage site.

The remaining 85 pairs of sRNA-targets with specific cleavage signals on the target genes passed the tests of the two analysis modules (“Forward Digger” and “Reverse Digger”) of sRNATargetDigger (S2 Table). As we expected, sRNATargetDigger found that the regulatory information reported in the literature [7] was incomplete, and the co-regulatory sRNAs were missed for 69 target genes in one or more tissues, and 170 novel sRNA-target pairs were identified (S2 Table). The regulatory pair "ath-miR156a-5p-AT1G27360.4" was taken as an example, co-regulatory sRNAs had been found in all tissues except the root, they had almost the same complementarities and binding sites with the target genes, and the levels of AGO1 proteins was even higher than that of ath-miR156a-5p. Among these novel regulatory sRNAs, four were found in flowers (sRNA_AT_38, sRNA_AT_39, sRNA_AT_40, sRNA_AT_17) and the other two (sRNA_AT_39, sRNA_AT_40) were found in leaves and seedlings (Fig 3).

As most of the newly discovered co-regulatory sRNAs have not been reported previously, their functions in regulating the targets need to be further confirmed by other credible methods. PAREsnip2 [13] was chosen to perform this confirmation and the 85 pairs of miRNA-targets supported by the literature [7] were re-analysed. As a result of this experiment, 144 out of 170 novel co-regulatory sRNA-target pairs identified by sRNATargetDigger passed the examination. For the control, 81 out of 85 miRNA-target pairs passed the test (S3 Table). Thus, the credibility of the regulatory relationship in these novel co-regulatory sRNA-target pairs was confirmed using PAREsnip2.

**Fig 4:**
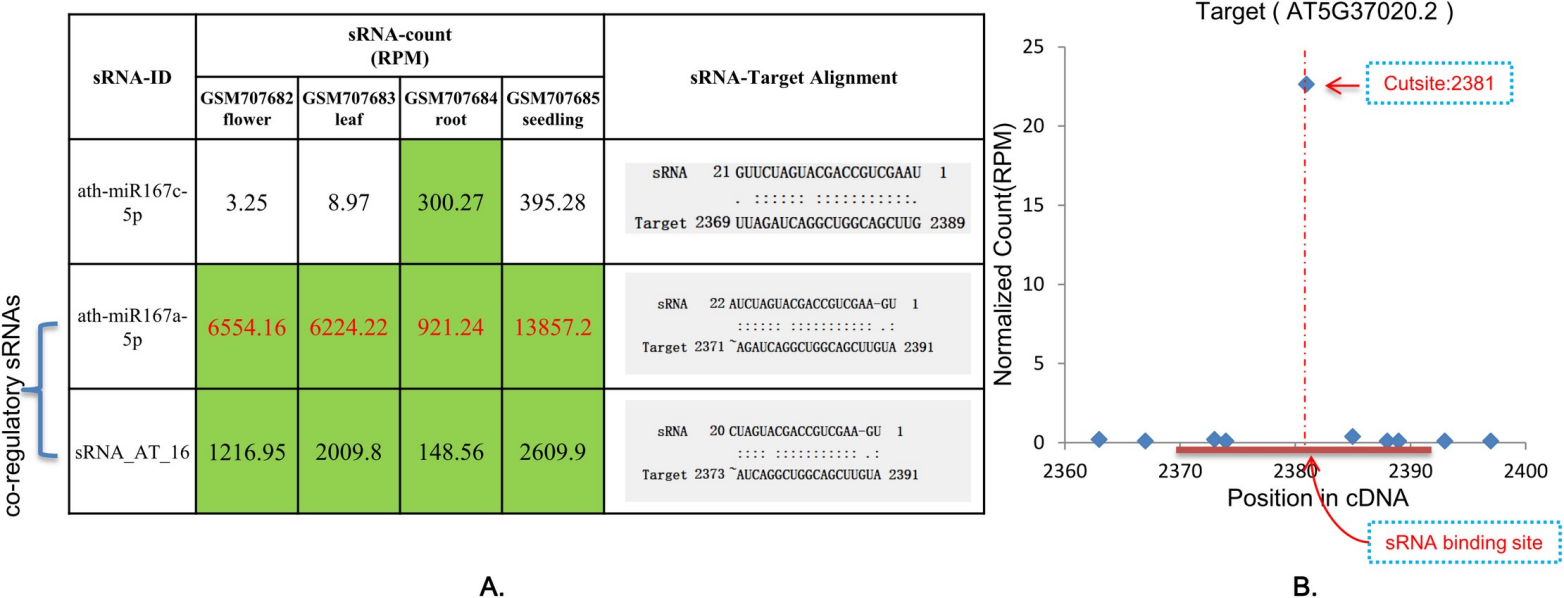
The miRNA ath-miR167c-5p and the co-regulating sRNAs with their target cDNA (AT5G37020.2) in *Arabidopsis*. A: Expression of ath-miR167c-5p and co-regulatory sRNAs in Arabidopsis flowers, leaves, roots and seedling and their alignments with target genes; green indicates that sRNA plays a regulatory role, white indicates that the regulatory role of the sRNA could be ignored, the red number indicates that the sRNA plays the most important regulatory role. B: T-plot map obtained after AT5G37020.2 and degradome matching.The abscissa indicates the position on the target gene, the ordinate indicates the intensity of the degradation signal, the red horizontal line indicates the sRNA binding site, and the red dotted line indicates the cleavage site.

Interestingly, a study has shown that some sRNA-target regulatory relationships collected from the literature [7] exhibit actually tissue-specific weak regulatory relationships. Taken "ath-miR167c-5p —> ARF8 (AT5G37020.2)" as an example, only in *Arabidopsis* roots, ath-miR167c-5p was found to be involved in the regulation of ARF8 as a low-expression co-regulatory sRNA. In the other three tissues (flowers, leaves and seedlings), its regulatory role was found to be negligible due to a relatively low-expression level, which cannot pass the “Forward Digger” test. When “Reverse Digger” was used to reversely search the regulatory sRNAs of ARF8, it was found that ath-miR167a-5p was the main regulator of ARF8. Its expression in *Arabidopsis* flowers, leaves, roots, and seedlings was very high, and had a good match with the target genes (Fig 4). This finding is consistent with the results of the transgenic experiment by Wu et al., which found that MIR167a (ath-miR167a-5p precursor) was the major functional miR167 precursor *in vivo*, whereas MIR167c (ath-miR167c-5p precursor) had a weak regulatory effect on ARF8 [29]. In addition, sRNATargetDigger found that sRNA_AT_16 also has a co-regulatory effect on ARF8 (Fig 4).

As we know, there are often many-to-many regulatory relationships in sRNAs-targets. When using the conventional degradome-based sRNA-target analysis software, the unknown co-regulatory sRNAs cannot be identified; thus, the obtained information is not comprehensive. The sRNATargetDigger by integrating with reverse mining technology, can solve this problem. The tests regarding sRNA-target in *Arabidopsis* indeed found several co-regulatory sRNAs that were highly complementary to the target genes, with binding sites almost identical to known sRNAs. Notably, their expression levels in some tissues even exceeded those of known sRNAs, indicating that they may play a more important regulatory role. Among them, we found that the miRNA family members with high sequence identity often regulated some common target genes; the relevant results agreed also with the literature. For example, ath-miR169a-5p and ath-miR169d have been shown to co-regulate NFYA2 (AT3G05690.1) [30, 31]; ath-miR172b and ath-miR172c to co-regulate AP2 (AT4G36920.1) [32, 33]; ath-miR396a-5p and ath-miR396b-5p to co-regulate the GRF family (AT2G22840.1, AT4G37740.1, AT2G36400.1, AT3G52910.1 and AT2G45480.1) [34]. Therefore, based on the degradome and sRNA HTS data, sRNATargetDigger can help researchers to build a more comprehensive and reliable sRNA-target regulatory relationship.

## Conclusions

Based on HTS data analysis, we developed a novel software named sRNATargetDigger that has two function modules “Forward Digger” and “Reverse Digger”, and this software can identify regulatory sRNA-target pairs bidirectionally. Compared to the other sRNA-target mining softwares, it has the ability to identify unknown sRNAs co-regulating the same target, so as to reveal a more comprehensive and reliable sRNA-target regulatory relationship. When the published sRNA-target pairs in *Arabidopsis* were re-examined, all the sRNA-targets with specific cleavage signals on the target genes passed the tests of sRNATargetDigger. Moreover, it identified 170 novel co-regulatory sRNA-target pairs. The developed pipeline can automatically complete the analysis only by inputting data. Therefore, this software may be a popular prediction tool for the plant biologists in sRNA-target research.

## Supporting information

S1 Table Targets with no cleavage signals detected using degradome in *Arabidopsis*. (XLS)

S2 Table "sRNA-target" pairs and their co-regulatory sRNAs identified using sRNATargetDigger in *Arabidopsis*. (XLS)

S3 Table Verification of the results on the regulatory relationship of novel co-regulatory sRNA-target pairs using PAREsnip2. (XLS)

## References

[1] Gregory RI, Kai-Ping Y, Govindasamy A, Thimmaiah C, Behzad D, Neil C, et al The Microprocessor complex mediates the genesis of microRNAs. *Nature*. 2004; 432(7014):235–240. 10.1038/nature03120 15531877

[2] Lewis BP, I-Hung S, Jones-Rhoades MW, Bartel DP, Burge CB. Prediction of mammalian microRNA targets. *Cell*. 2003;115(7):787–798. 10.1016/s0092-8674(03)01018-3 14697198

[3] Nicolas FE. Experimental Validation of MicroRNA Targets Using a Luciferase Reporter System. *Methods Mol Biol*. 2011;732:139–152. 10.1007/978-1-61779-083-6_11 21431711

[4] Xue C, Li F, He T, Liu GP, Li Y, Zhang X. Classification of real and pseudo microRNA precursors using local structure-sequence features and support vector machine. *Bmc Bioinformatics*. 2005;6(1):310 10.1186/1471-2105-6-310 16381612 PMC1360673

[5] Friedlander MR, Mackowiak SD, Na L, Wei C, Nikolaus R. miRDeep2 accurately identifies known and hundreds of novel microRNA genes in seven animal clades. *Nucleic Acids Research*. 2012;40(1):37–52. 10.1093/nar/gkr688 21911355 PMC3245920

[6] Michael H, Naiara RE, Aransay AM. miRanalyzer: an update on the detection and analysis of microRNAs in high-throughput sequencing experiments. *Nucleic Acids Research*. 2011;39(Web Server issue):W132–138. 10.1093/nar/gkr247 21515631 PMC3125730

[7] Dai X, Zhuang Z, Zhao PX. psRNATarget: a plant small RNA target analysis server (2017 release). *Nucleic Acids Research*. 2018;46(W1):W49–W54. 10.1093/nar/gky316 29718424 PMC6030838

[8] German MA, Manoj P, Dong-Hoon J, Amit H, Shujun L, Prakash J, et al Global identification of microRNA-target RNA pairs by parallel analysis of RNA ends. *Nature Biotechnology*. 2008;26(8):941–946. 10.1038/nbt1417 18542052

[9] Gregory BD, O'Malley RC, Lister R, Urich MA, Tonti-Filippini J, Chen H, et al A Link between RNA Metabolism and Silencing Affecting Arabidopsis Development. *Developmental Cell*. 2008;14(6):854–866. 10.1016/j.devcel.2008.04.005 18486559

[10] Addo-Quaye C, Eshoo TW, Bartel DP, Axtell MJ. Endogenous siRNA and miRNA Targets Identified by Sequencing of the Arabidopsis Degradome. *Current Biology*. 2008;18(10):758–762. 10.1016/j.cub.2008.04.042 18472421 PMC2583427

[11] Addo-Quaye C, Miller W, Axtell MJ. CleaveLand: a pipeline for using degradome data to find cleaved small RNA targets. *Bioinformatics*. 2009;25(1):130–131. 10.1093/bioinformatics/btn604 19017659 PMC3202307

[12] Zheng Y, Li YF, Sunkar R, Zhang W. SeqTar: an effective method for identifying microRNA guided cleavage sites from degradome of polyadenylated transcripts in plants. *Nucleic Acids Research*. 2011;40(4):e28 10.1093/nar/gkr1092 22140118 PMC3287166

[13] Thody J, Folkes L, Medinacalzada Z, Xu P, Dalmay T, Moulton V. PAREsnip2: A tool for high-throughput prediction of small RNA targets from degradome sequencing data using configurable targeting rules. *Nucleic Acids Research*. 2018; 46(17):8730–8739. 10.1093/nar/gky609 30007348 PMC6158750

[14] Kakrana A, Hammond R, Patel P, Nakano M, Meyers BC. sPARTA: a parallelized pipeline for integrated analysis of plant miRNA and cleaved mRNA data sets, including new miRNA target-identification software. *Nucleic Acids Research*. 2014;42(18):e139 10.1093/nar/gku693 25120269 PMC4191380

[15] Zhu H, Zhang Y, Tang R, Qu H, Duan X, Jiang Y. Banana sRNAome and degradome identify microRNAs functioning in differential responses to temperature stress. *BMC Genomics*. 2019;20(1) 33 10.1186/s12864-018-5395-1 30630418 PMC6329063

[16] Huen A, Bally J, Smith P. Identification and characterisation of microRNAs and their target genes in phosphate-starved Nicotiana benthamiana by small RNA deep sequencing and 5’RACE analysis. *BMC Genomics*. 2018;19(1):940 10.1186/s12864-018-5258-9 30558535 PMC6296076

[17] Luria N, Smith E, Reingold V, Bekelman I, Lapidot M, Levin I, et al A New Israeli Tobamovirus Isolate Infects Tomato Plants Harboring Tm-22 Resistance Genes. *Plos One*. 2017;12(1):e0170429 10.1371/journal.pone.0170429 28107419 PMC5249172

[18] Mutum RD, Kumar S, Balyan S. Identification of novel miRNAs from drought tolerant rice variety Nagina 22. *Sci Rep*. 2016;6(1):30786 10.1038/srep30786 27499088 PMC4976344

[19] Bartel DP. MicroRNAs: Target Recognition and Regulatory Functions. *Cell*. 2009;136(2):215–233. 10.1016/j.cell.2009.01.002 19167326 PMC3794896

[20] Shao C, Chen M, Meng Y. A reversed framework for the identification of microRNA-target pairs in plants. *Briefings in Bioinformatics*. 2013;14(3):293–301. 10.1093/bib/bbs040 22811545

[21] Allen E, Xie Z, Gustafson A JC. microRNA-directed phasing during trans-acting siRNA biogenesis in plants. *Cell*. 2005;121(2):207–221. 10.1016/j.cell.2005.04.004 15851028

[22] Fahlgren N, Carrington JC. miRNA Target Prediction in Plants. *Methods Mol Biol*. 2010;592:51–57. 10.1007/978-1-60327-005-2_4 19802588

[23] Axtell MJ. Classification and Comparison of Small RNAs from Plants. *Annual Review of Plant Biology*. 2013;64(1):137–159. 10.1146/annurev-arplant-050312-120043 23330790

[24] Barturen G, Rueda A, Hamberg M, Alganza A, Lebron R, Kotsyfakis M, et al sRNAbench: profiling of small RNAs and its sequence variants in single or multi-species high-throughput experiments. *Methods in Next Generation Sequencing*. 2014; 1:21–31.

[25] Ma Z, Hu X, Cai W, Huang W, Zhou X, Luo Q, et al Arabidopsis miR171-targeted scarecrow-like proteins bind to GT cis-elements and mediate gibberellin-regulated chlorophyll biosynthesis under light conditions.*PLoS genetics*. 2014;10(8): e1004519 10.1371/journal.pgen.1004519 25101599 PMC4125095

[26] Naya L, Paul S, Valdés-López O, Mendoza-Soto AB, Nova-Franco B, Sosa-Valencia G, et al Regulation of copper homeostasis and biotic interactions by microRNA 398b in common bean. PloS one. 2014; 9(1): e84416 10.1371/journal.pone.0084416 24400089 PMC3882225

[27] Ye X, Jiang Y, Yu L, Yang Z, Meng Y, Shao C. Identification of novel microRNAs in rice (Oryza sativa) based on the cleavage signals in precursors. *Plant Biosystems*. 2019;153(4):506–513.

[28] Yu L, Shao C, Ye X, Meng Y, Zhou Y, Chen M. miRNA Digger: a comprehensive pipeline for genome-wide novel miRNA mining. *Scientific Reports*. 2016;6:18901 10.1038/srep18901 26732371 PMC4702050

[29] Miin-Feng W, Qing T, Reed JW. Arabidopsis microRNA167 controls patterns of ARF6 and ARF8 expression, and regulates both female and male reproduction. *Development*. 2006;133(21):4211–4218. 10.1242/dev.02602 17021043

[30] Hanna L, Jeon YS, Hwan LJ, Wanhui K, Kwan YS, Heather F, et al Genetic framework for flowering-time regulation by ambient temperature-responsive miRNAs inArabidopsis. *Nucleic Acids Research*. 2010;38(9):3081–3093. 10.1093/nar/gkp1240 20110261 PMC2875011

[31] Xu MY, Zhang L, Li WW, Hu XL, Wang MB, Fan YL, et al Stress-induced early flowering is mediated by miR169 in Arabidopsis thaliana. *Journal of Experimental Botany*. 2014;65(1):89–101. 10.1093/jxb/ert353 24336445

[32] Kasschau KD, Xie Z, Allen E, Llave C, Chapman EJ, Krizan KA, et al P1/HC-Pro, a Viral Suppressor of RNA Silencing, Interferes with Arabidopsis Development and miRNA Function. *Developmental Cell*. 2003;4(2):205–217. 10.1016/s1534-5807(03)00025-x 12586064

[33] Xuemei C. A microRNA as a translational repressor of APETALA2 in Arabidopsis flower development. *Science*. 2004;303(5666):2022–2025. 10.1126/science.1088060 12893888 PMC5127708

[34] Liu D, Song Y, Chen Z, Yu D. Ectopic expression of miR396 suppresses GRF target gene expression and alters leaf growth in Arabidopsis. *Physiologia Plantarum*. 2009;136(2):223–236. 10.1111/j.1399-3054.2009.01229.x 19453503

